# Bottom-Up Synthesis of De-Functionalized and Dispersible Carbon Spheres as Colloidal Adsorbent

**DOI:** 10.3390/ijms24043831

**Published:** 2023-02-14

**Authors:** Maria Balda, Katrin Mackenzie, Silke Woszidlo, Hans Uhlig, Jens Möllmer, Frank-Dieter Kopinke, Gerrit Schüürmann, Anett Georgi

**Affiliations:** 1 Department of Environmental Engineering, Helmholtz Centre for Environmental Research—UFZ, 04318 Leipzig, Germany; 2 Institut für Nichtklassische Chemie e.V.—INC, 04318 Leipzig, Germany; 3 Institute of Organic Chemistry, Technical University Bergakademie Freiberg, 09599 Freiberg, Germany; 4 Department of Ecological Chemistry, Helmholtz Centre for Environmental Research—UFZ, 04318 Leipzig, Germany

**Keywords:** activated carbon spheres, dispersibility, hydrothermal carbonization, adsorption, groundwater remediation, colloidal activated carbon

## Abstract

Recent innovative adsorption technologies for water purification rely on micrometer-sized activated carbon (AC) for ultrafast adsorption or in situ remediation. In this study, the bottom-up synthesis of tailored activated carbon spheres (aCS) from sucrose as renewable feedstock is demonstrated. The synthesis is based on a hydrothermal carbonization step followed by a targeted thermal activation of the raw material. This preserves its excellent colloid properties, i.e., narrow particle size distribution around 1 µm, ideal spherical shape and excellent aqueous dispersibility. We investigated the ageing of the freshly synthesized, highly de-functionalized AC surface in air and aqueous media under conditions relevant to the practice. A slow but significant ageing due to hydrolysis and oxidation reactions was observed for all carbon samples, leading to an increase of the oxygen contents with storage time. In this study, a tailored aCS product was generated within a single pyrolysis step with 3 vol.-% H_2_O in N_2_ in order to obtain the desired pore diameters and surface properties. Adsorption characteristics, including sorption isotherms and kinetics, were investigated with monochlorobenzene (MCB) and perfluorooctanoic acid (PFOA) as adsorbates. The product showed high sorption affinities up to log (*K*_D_/[L/kg]) of 7.3 ± 0.1 for MCB and 6.2 ± 0.1 for PFOA, respectively.

## 1. Introduction

Activated carbon (AC) with a particle size of about 1 µm has recently gained increasing attention as so-called super-fine powdered activated carbon (SPAC) or colloidal AC. Compared to conventional powdered activated carbon (PAC) in the size range of 10–100 µm, SPAC typically provides very fast adsorption kinetics, which is a necessary adsorbent property for efficient wastewater treatment [1,2]. Furthermore, colloidal activated carbon is already applied at a large scale in the field of in situ groundwater remediation [3,4,5]. In this case, the suspended particles are injected directly into the contaminated aquifer. There, they are deposited on the aquifer sediment and form a permeable sorption barrier in order to retain pollutants and prevent them from further spreading with the groundwater flow. In situ groundwater remediation is often the only way to provide damage control at polluted sites that are difficult to access or where the remediation via pump-and-treat of polluted water would be too expensive and time-consuming. One of the major environmental problems we are currently facing are per- and polyfluorinated aliphatic substances (PFAS). Various PFAS congeners are known to have adverse-health effects such as endocrine disruption [6,7]. Due to their high mobility and simultaneous persistency, these so-called “forever chemicals” are a serious environmental issue [8]. Regarding PFAS, the remediation with well-adapted colloidal AC is currently the only in situ treatment option applied at the technical scale for efficient damage mitigation [9,10].

For the production of SPAC, commercially available PAC is crushed to a mean particle size close to 1 µm in a wet-milling process with a duration of several hours [2,11]. In this work, an alternative synthesis strategy for a SPAC-like product was developed and denominated as activated carbon spheres (aCS). aCS particles in the desired size-range were generated bottom-up via the hydrothermal carbonization (HTC) of sucrose with subsequent activation under pyrolysis conditions.

To obtain the desired product, we had to solve the following research questions:

1. How can the dispersibility of the individual particles be maintained during pyrolysis?
2. How can the pore size distribution and surface chemistry of the aCS be tuned during the pyrolysis step?
3. How does the surface of the synthesized aCS change over time, and how can it be stabilized?

As mentioned in a former publication [12], the dispersibility of HTC-generated carbonaceous spheres (CS), especially after pyrolysis, has not yet been closely investigated but was merely mentioned in passing [13,14]. However, for designing the desired product, its dispersibility is a crucial property and can too easily be erased by choosing unfavorable pyrolysis conditions. Therefore, the impact of the pyrolysis process on the product quality was intensively investigated within this work. A low heating rate was identified as the deciding parameter for maintaining the particle dispersibility. To our knowledge, this has not been described in the literature before. Furthermore, for the in situ application, the behavior of the aCS in aqueous suspensions over time is important. The sedimentation and/or agglomeration were monitored in aqueous suspension for the synthesized aCS in comparison with a finely ground commercial AC sample.

Due to the presence of water and the high energy input during the milling process, the surface of the AC particles is prone to be oxidized. It was found that this decreases the adsorption capacities for non-ionic organic compounds [15]. This is thought to have an even greater impact on more challenging substances such as perfluorooctanoic acid (PFOA), which has a highly hydrophobic chain and an anionic head group. It was recently shown that even a slight oxidation of the AC surface could have a negative influence on the sorption performance [16]. With the herein developed bottom-up synthesis, aCS properties, e.g., pore size distribution and surface functional groups, can be tuned by choosing suitable parameters during the pyrolysis/activation step.

Monitoring the aCS in aqueous suspensions eventually led us to our last research questions: (i) how fast does the particle surface age under relevant conditions regarding storage and application, and (ii) what are the possible underlying mechanisms? Some mechanisms have been studied regarding AC surfaces, e.g., for the role of water vapor in activated carbon ageing at 150 °C [17]. However, to the best of our knowledge, there are no studies that investigate the surface oxidation of (pyro-)hydrochar under ambient conditions in water or air. Furthermore, for de-functionalized AC surfaces, additional treatment with hydrogen gas might be necessary in order to maintain very low oxygen contents of the surface [18]. As an additional high-temperature treatment with hydrogen is energy-consuming and safety-relevant, we developed a pyrolysis procedure where the desired porosity as well as de-functionalization and stabilization of the surface were achieved directly within one pyrolysis step. For potential applications in wastewater treatment and in situ aquifer remediation, changes of the adsorbent surface upon storage/application in water are crucial. This applies especially when it comes to more demanding pollutants such as PFOA, which favors a very hydrophobic carbon sorbent surface. Therefore, we further investigated the ageing of our synthesized aCS in oxygen-containing water and air of different humidities over 30 days. In order to characterize their performance as colloidal adsorbents, the sorption of monochlorobenzene (MCB) as well as PFOA was studied in more detail.

## 2. Results and Discussion

### 2.1. Maintaining the Excellent Dispersibility of Carbon Spheres after the Pyrolysis Step

**Figure 1:**
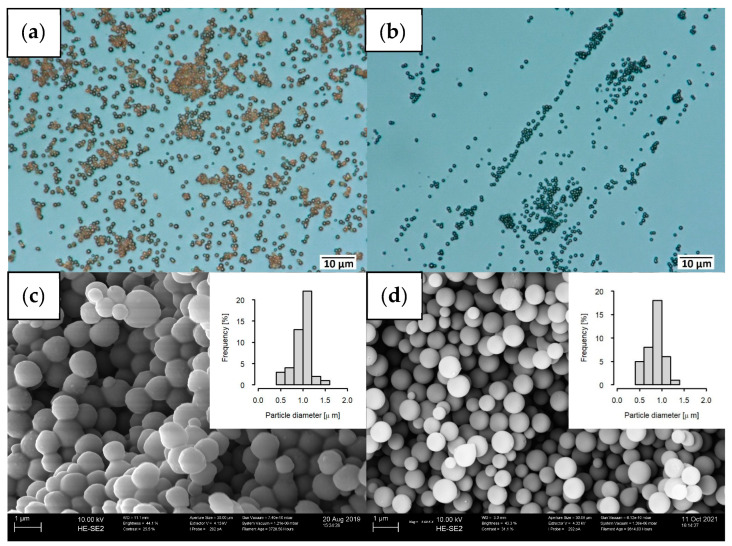
(**a**) Digital microscopy of carbon spheres (CS) and (**b**) activated CS (aCS); (**c**) SEM of CS and (**d**) aCS; histograms of the particle distribution were obtained through measurement of 100–150 particles (ImageJ) and data evaluation (RStudio).

When the CS generated via HTC were pyrolyzed under a nitrogen atmosphere, they were further carbonized and developed a microporous structure (cf. Table 1). Upon carbonization, the CS changed their color from brown to black (Figure 1a,b) and appeared to be hardened (Figure 1, bottom). The particles’ mean diameters did not change significantly. In order to find the optimal heating scheme to maintain the dispersibility in water of the HTC-derived CS, three different heating rates—100 K min^−1^, 10 K min^−1^ and 1 K min^−1^—were investigated. After the pyrolysis at 600 °C for 1 h under N_2_, the different samples were added into water, shaken for at least 24 h and then treated with sonication for 10 min.

**Figure 2:**
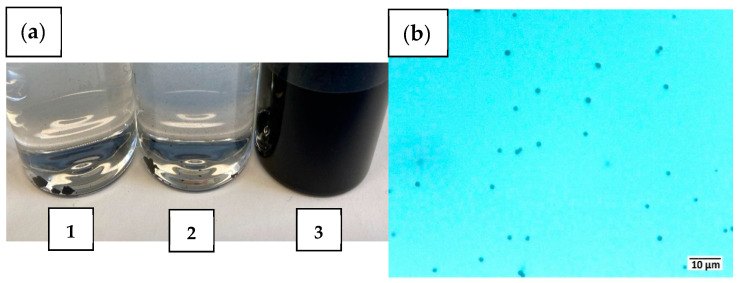
(**a**) CS pyrolyzed at 600 °C for 1 h under N_2_ with heating rates of (**1**) 100, (**2**) 10 and (**3**) 1 K min^−1^, respectively (with (**1**) and (**2**) showing large non-dispersible aggregates at the bottom); and (**b**) digital microscopy of sample (**3**).

In Figure 2a, the drastic differences between the three differently heated samples are shown. The CS samples, which were heated with 100 or 10 K min^−1^, could not be dispersed into individual particles. Instead, the particles remain predominantly as large aggregates on the bottom of the flask (Figure 2a, vials 1 and 2). In contrast, the sample that was obtained with very slow heating (1 K min^−1^) was well dispersible, forming a homogeneous and stable black dispersion. That the primary particle size of the CS was preserved in the latter sample was confirmed through microscopic analysis, shown in Figure 2b. The particle size distribution in the aqueous suspension remained monomodal. It was measured by dynamic light scattering (Figure S3) and exhibited a Gaussian distribution with a mean value of (1.02 ± 0.03) µm, which is consistent with the *d*_90_ of 1.04 µm obtained from particle characterization in the dry state.

For understanding the effect of the heating rate on the dispersibility of the CS, the mechanisms behind pyrolysis processes have to be considered. At low temperatures (<260 °C), mainly dehydration reactions occur, which can proceed either in an intra-particulate or inter-particulate way. At higher temperatures (>400 °C), condensation and aromatization processes are favored, which can lead to further crosslinking [19]. Intra-particulate condensation and aromatization lead to the solidification of the individual lignite-like hydrochar particles, whereas the respective inter-particulate reactions may result in the aggregation of particles. As a result, lower heating rates favor higher degrees of solidification of “visco-plastic” particles prior to cross-linking reactions between particles at higher temperatures. Solid particles may have less contact areas compared to visco-plastic particles.

### 2.2. Tuning of Surface Properties and Mean Pore Diameters during the Pyrolysis Step

In order to understand the influence of the pyrolysis parameters on the pore structure as well as the surface of the synthesized CS, an extensive set of experiments was performed, where the pyrolysis temperature, gas atmosphere and holding time were varied. The resulting CS samples were characterized by temperature-programmed decomposition (TPD) as well as CO_2_ ad-/desorption measurements. The samples pyrolyzed under N_2_ showed a decrease in oxygen content with an increasing pyrolysis temperature from 8.8 wt.-% oxygen (pCS400) over 2.3 wt.-% O (pCS600) to 1.3 wt.-% O (pCS800). This effect is generally known in the literature and was recently shown for the pyrolysis of hydrochars derived from cellulose and woody biomass [20]. For these types of hydrochars, the oxygen contents typically decreased from 25–30 wt.-% to 14 wt.-% at pyrolysis temperatures of 400 and 600 °C, respectively.

**Figure 3:**
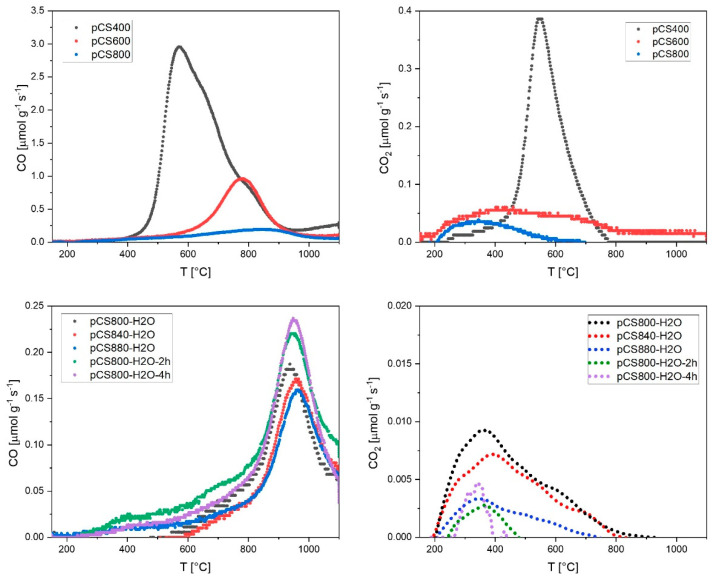
CO and CO_2_ release from temperature-programmed decomposition (TPD) up to 1100 °C (10 K min^−1^) of all pyrolyzed CS samples; CO_2_ thermograms of the steam-activated samples (bottom right) were smoothed via locally weighted scatterplot smoothing (LOWESS) with a smoothing parameter of 0.1.

In order to investigate the type of functional groups more closely, the temperature-resolved TPD profiles are shown in Figure 3. The total content of the oxygen functional groups was decreased as thermally labile groups were detached during the pyrolysis at higher temperatures, leaving behind only the functional groups with higher thermal stabilities. Therefore, the major CO peak shifted to higher temperatures for materials that were pyrolyzed at higher temperatures under N_2_ (Figure 3, top left). According to the assignment of T-dependent CO release from O-groups on carbon surfaces presented by Figueiredo et al. [21], this means that the major amount of CO-releasing functional groups was shifted from α-substituted carbonyls/aldehydes to aromatic carbonyls/quinones and/or phenols. The major peak in the CO_2_ thermogram of pCS400 could be assigned to anhydride groups. It can be observed that after the pyrolysis at temperatures > 600 °C, anhydride groups are almost completely annihilated and a small amount of free carboxylic groups remains as the only CO_2_-releasing functional groups on the pCS surface, probably due to re-oxidation after pyrolysis.

The de-functionalization effect of the conducted pyrolysis was significantly enhanced when 3 vol.-% H_2_O was added to the inert pyrolysis atmosphere. The overall TPD-derived oxygen contents decreased from (2.6 ± 0.2) wt.-% for pCS800 to as low as (0.5 ± 0.2) wt.-% for pCS800-H_2_O.

Furthermore, Figure 3 shows that the de-functionalization of the carbon surface was more pronounced at higher temperatures. The CO and CO_2_ release decreased from pCS800-H_2_O over pCS840-H_2_O to pCS880-H_2_O. Longer reaction times further reduced the CO_2_ release but also led to a slight increase in CO release again, resulting in slightly higher TPD-derived O-contents of (0.9 ± 0.2) wt.-% for pCS800-H_2_O-2h and (1.2 ± 0.2) wt.-% for pCS800-H_2_O-4h.

The CS pyrolyzed under different conditions all provide a strictly microporous system with pore sizes < 1 nm (see Figure S2). The distinct main pore fraction of all samples exhibits diameters of around 0.5–0.7 nm. Only small differences between all CS samples could be monitored regarding the mean pore diameter. CS pyrolyzed under pure N_2_ showed an increase of the C content as well as of the CO_2_-sorption-derived surface area with an increasing pyrolysis temperature. Overall, the pore volume in the size range of 0.5 to 0.7 nm increased with increasing pyrolysis temperature and, in addition, a larger contribution of even smaller pores appeared (Figure S2). In contrast, pyrolysis under 3 vol.-% H_2_O/N_2_ at three different temperatures (800, 840 and 880 °C) led to a significant widening of the mean pore diameter from 0.58 nm over 0.63 nm to 0.68 nm, respectively. It is known from the literature that steam can widen the microporosity of carbonaceous materials, as was shown for the activation of carbonized olive stones [22,23]. It is noteworthy that the steam concentration of 3 vol.-% that was applied in this work is low compared to typical dosages of 70 vol.-% used in literature studies [24]. Nevertheless, it had a significant effect on the resulting carbonaceous material regarding the additionally generated porosity and the widening of pores as well as on the surface de-functionalization as discussed above. A similar effect was found by Mestre et al., who generated activated carbon with basic properties from the activation of sucrose-derived hydrochar with 40 vol.-% steam [25]. In contrast to the pyrolysis temperature, the holding time up to 4 h at 800 °C had a less significant impact on the mean pore size.

**Figure 4:**
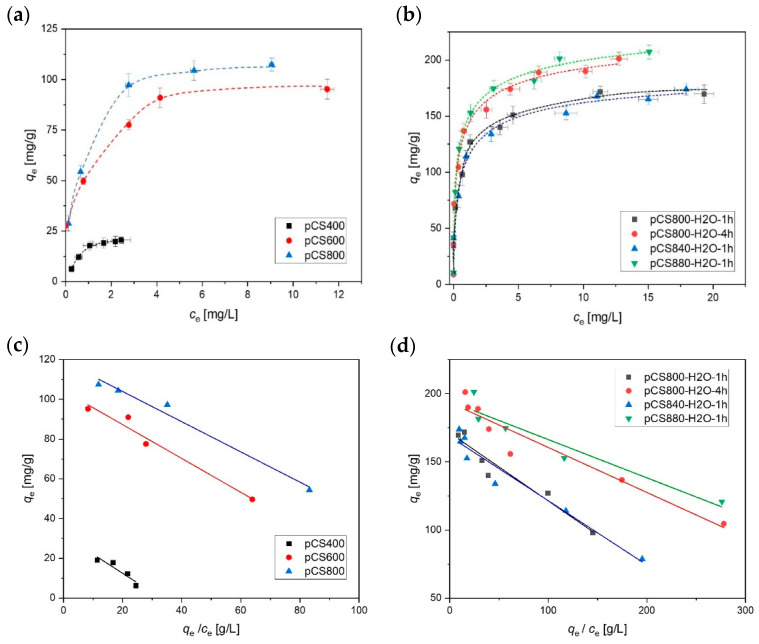
Isotherms of monochlorobenzene (MCB) sorption on hydrochar samples pyrolyzed under N_2_ (**a**,**c**) or under H_2_O/N_2_ (**b**,**d**). Error bars represent the mean deviation of single values from the mean value based on the experimental and analytical duplicates (i.e., n = 4). The dotted lines are a guide for the eye and the solid lines are fitted according the linearized Langmuir model. The results are summarized in Table 2. *c*_MCB_ = 2.5–30 mg L^−1^, *c*_pCS_ = 100 mg L^−1^, 10 mM KNO_3_, pH = 6.5.

In the first step, the sorption performance of the differently pyrolyzed samples was characterized by determining the adsorption of MCB as a non-ionic compound and frequently occurring groundwater contaminant (Figure 4). The experimentally obtained adsorption isotherms were evaluated and fitted according to the Langmuir model (Equation (1), Figure 4 bottom). The results are shown in Table 2. The steam-activated samples showed the highest sorption affinities (indicated by the highest *K*_L_ values). It is noteworthy that the sample obtained at a moderate pyrolysis temperature of 600 °C (pCS600) already exhibits rather good adsorption properties for MCB, which are not significantly improved by further increasing the pyrolysis temperature under N_2_ atmosphere. In contrast, activation with steam with a 1 h holding time at 800 °C (pCS800-H_2_O) significantly improved the adsorption compared to sample pCS800.

The maximum loading as well as *K*_L_ of the synthesized adsorbents could be gradually increased either with activation at higher temperatures or prolonging of the holding time. The sample pCS800-H_2_O-4h, called “activated carbon spheres” = aCS, was selected for further thorough characterization.

**Figure 5:**
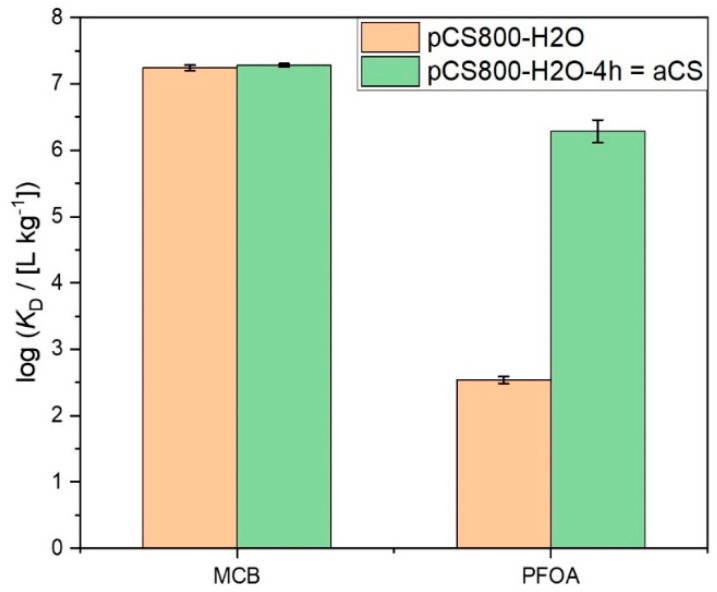
Single point adsorption coefficients log *K*_D_ on pCS800-H_2_O vs. aCS for MCB (*c*_0,MCB_ = 2.5 mg L^−1^; *c*_e,MCB_ = 2 µg L^−1^; *c*_aCS_ = 70 mg L^−1^; 10 mM KNO_3_; 6 < pH < 7) and PFOA (*c*_0,PFOA_ = 0.5 mg L^−1^; *c*_e,PFOA_ = 2 µg L^−1^; *c*_aCS_ = 200 mg L^−1^; 10 mM NaCl; pH = 6.5). Error bars represent the mean deviation of single values from the mean value of at least two independent experiments.

When shifting from the small target molecule MCB to PFOA, the impact of the activation step on the accessibility of the CS pores for adsorption became more obvious. For chain-like molecules such as PFOA, the effective diameter *D*_eff_, i.e., the cross-sectional diameter at maximum elongation of the molecule is relevant to evaluate its fit into the CS pores. In order to determine this parameter we applied the calculation tool CROSS [26] CROSS is a program for the approximate calculation of the effective largest, effective second largest (=effective) and effective smallest diameters of three-dimensional Cartesian coordinate representations of molecules. The effective largest diameter *D*_max_ is defined such that the respective perpendicular diameter is minimal, the latter being the effective second largest or simply effective diameter *D*_eff_; the effective third largest diameter *D*_min_ is the diameter perpendicular to both *D*_max_ and *D*_eff_. The resulting value of *D*_eff_ = 0.6 nm for PFOA is in good agreement with literature findings [27] and fits well with the experimentally determined mean pore diameter of aCS of 0.6 nm (cf. Table 1). Even though there was nearly no change in the pore size distribution apparent between the two particle types activated with 1 and 4 h holding time, i.e., pCS800-H_2_O and aCS (Figure S2), and although a slight tendency towards a larger mean pore diameter was observed, the longer activation time for aCS drastically improved PFOA adsorption, as shown in Figure 5. The single-point adsorption coefficient was increased by four orders of magnitude from log (*K*_D,PFOA_/(L kg^−1^)) = 2.6 to 6.6 for aCS.

Therefore, it is assumed that on pCS800-H_2_O, PFOA can merely adsorb on the outer surface of the spherical particles, while on aCS, PFOA molecules are able to enter the microporous system. This assumption was supported by calculating the theoretically possible surface coverage of the particulate adsorbents with PFOA molecules (see Supporting Information). 

**Figure 6:**
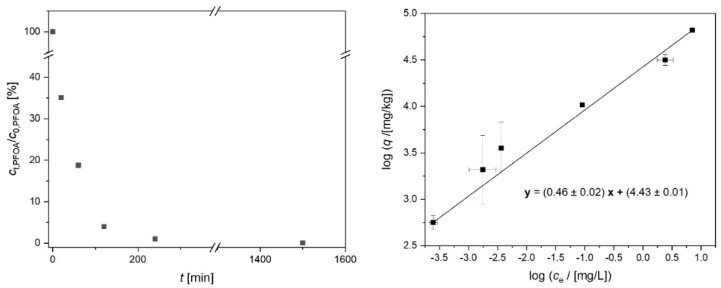
Time-resolved perfluorooctanoic acid (PFOA) adsorption (**left**) as well as the linearized adsorption isotherm (**right**) according to the Freundlich model (Equation (2)) on aCS. Error bars represent the minimum and maximum deviation from the mean value based on the experimental and analytical duplicates (i.e., n = 4). *c*_0,PFOA,kinetics_ = 0.2 mg L^−1^; *c*_0,PFOA,isotherm_ = 0.2–20 mg L^−1^, *c*_aCS_ = 200 mg L^−1^, 10 mM NaCl, pH = 6.5.

In order to elucidate the strong effect of the close tailoring of the pore diameter on the adsorption of a target compound, the adsorption of PFOA was investigated in more detail regarding kinetics as well as thermodynamics (Figure 6). Most of the PFOA is adsorbed within a few hours. This can be expected for porous adsorbents in the microscale. Regarding the in situ application of aCS particles as adsorbents in groundwater, the PFOA adsorption kinetics can be considered sufficiently fast due to typically low groundwater velocities of below 1 m d^−1^ [11]. The adsorption isotherm of PFOA on the microporous aCS was fitted according to the linearized Freundlich model (Equation (2)) with R^2^ = 0.995. The obtained adsorption parameters log (*K*_F_/[(mg kg^−1^)/(mg L^−1^)^1/n^]) = 4.43 and *n^−^*^1^ = 0.46 are comparable with literature data for PFOA adsorption on AC. For example, Wang et al. (2015) determined log *K*_F_ = 4.48 and *n^−^*^1^ = 0.43 for PFOA adsorption on microporous AC fibers with a mean pore diameter of 0.56 nm and a BET surface area of 1226 m^2^ g^−1^. The obtained low *n^−^*^1^ values reflect strongly non-linear sorption isotherms, i.e., a spectrum of high-affinity sorption sites. The maximum adsorption capacity of aCS was not yet reached at an equilibrium PFOA concentration of *c*_e_,_PFOA_ = 7.1 mg L^−1^ and loading of *q*_e,PFOA_ = 66,000 mg kg^−1^. 

### 2.3. Suspension Stability of the Activated Carbon Spheres

**Figure 7:**
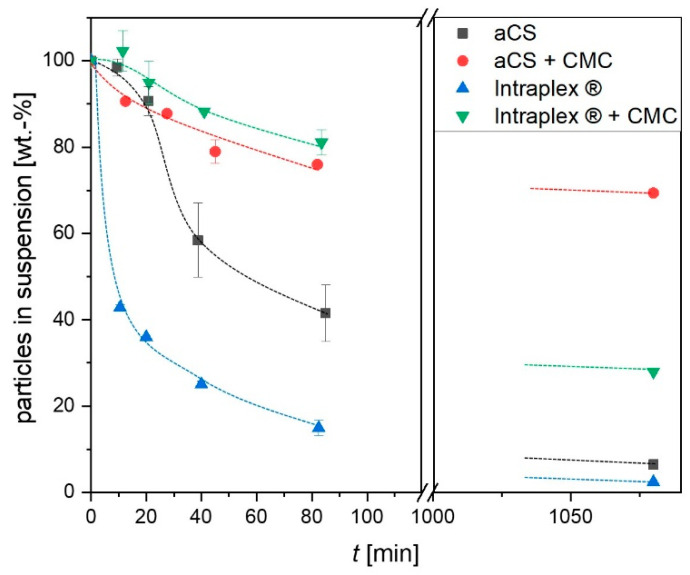
Sedimentation of the synthesized aCS in aqueous suspension in the absence and in the presence of CMC in comparison with a commercial finely powdered activated carbon (AC, Intraplex^®^). Dotted lines are a guide for the eye. Error bars represent the mean deviation of single values from the mean value of at least two independent experiments.

Considering the possible future in situ application for the synthesized aCS, the aqueous suspension properties of the particles are crucial and therefore were carefully investigated. The sedimentation of the particles in an aqueous suspension was monitored over 18 h in comparison with a commercial colloidal activated carbon (Intraplex^®^). When the aCS as well as Intraplex^®^ particles were not stabilized with carboxymethyl cellulose (CMC), they showed agglomeration and relatively fast sedimentation of the formed agglomerates. However, due to their perfectly spherical shape, the aCS particles were less prone to agglomeration compared to the commercial sample (Intraplex^®^), as shown in Figure 7. When stabilized with CMC, both the herein synthesized and the commercial particles sedimented slowly as mostly individual particles and stayed in the suspension much longer than without CMC. The effect can be attributed to an introduction of a surface charge by CMC sorption onto the external particle surface and the resulting electro-sterical stabilization effect [28].

**Figure 8:**
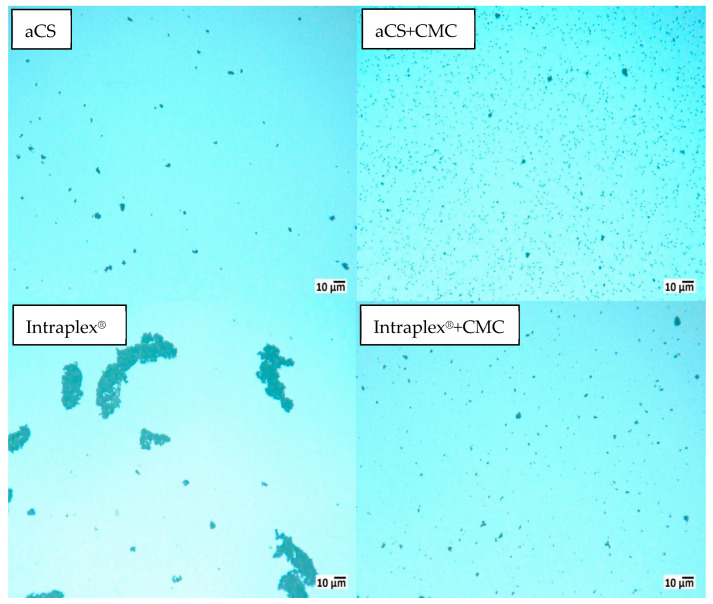
The 1 g L^−1^ AC particles in 10 mM KNO_3_ after >18 h in non-agitated suspensions in the presence and in the absence of 10 wt.-% carboxymethyl cellulose (CMC) (relating to the AC particles).

This was confirmed by zeta potential measurements, which showed decreases from (−31.7 ± 3.6) mV for aCS to (−45.5 ± 1.0) mV for aCS + CMC and from (−35.1 ± 0.8) mV for Intraplex^®^ to (−40.0 ± 2.5) mV for Intraplex^®^ + CMC. Here as well, the aCS were stable for a longer time. After storage for 18 h in a non-agitated state, 70 wt.-% of aCS + CMC remained in suspension while only 28 wt.-% of Intraplex^®^ + CMC were still suspended. On the one hand, this can be explained by the lower zeta potential of aCS + CMC compared to the commercial sample stabilized with CMC. On the other hand, the commercial finely ground particles are not spherical and thus provide a greater external surface for adhesive forces when particles come into contact. Furthermore, the rougher surface of the ground particles counteracts the shearing forces induced by the Brownian motion of the particles and consequently hinders their separation. This enhanced agglomeration of the commercial AC sample (in the absence of stabilizing CMC) can be impressively viewed under the microscope (Figure 8, sample “Intraplex^®^”).

### 2.4. Ageing of the Activated Carbon Spheres

**Figure 9:**
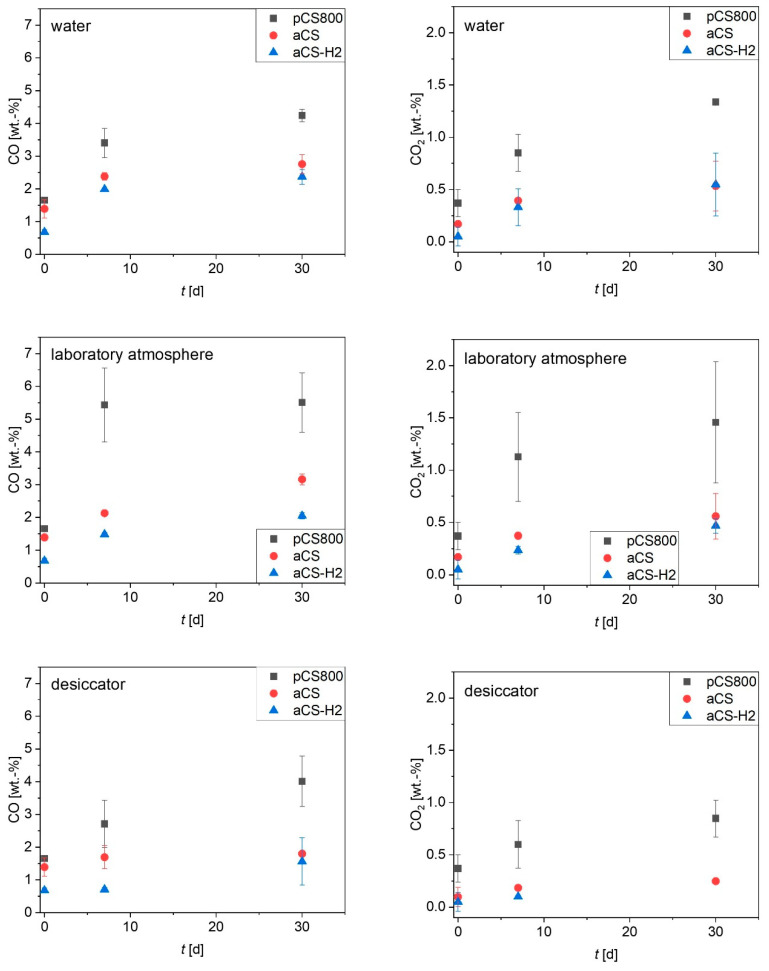
CO and CO_2_ released during TPD up to 1100 °C of the samples after various storage times t as an indicator of the amount of functional groups on the carbon surface. Error bars represent the mean deviation of single values from the mean value of at least two independent experiments. The titles of the diagrams represent the ageing conditions of the freshly synthesized aCS samples.

The oxygen content of the carbon surface is important for surface hydrophobicity and charge, and is thus a crucial factor for adsorption performance, especially towards ionic organic compounds [16,29]. The surface ageing of aCS (pyrolyzed in 3 vol.-% H_2_O/N_2_) over time was monitored in comparison with the ageing processes of pCS800 (pyrolyzed in dry N_2_) and aCS-H_2_ (aCS treated with 10 vol.-% H_2_/N_2_ at 900 °C). From Figure 9, it becomes evident that all samples age fastest when being stored in open beakers under a laboratory atmosphere and slowest in the desiccator. Sample pCS800, which was pyrolyzed under only N_2_, aged to the highest extent, which could not be prevented even by storage under a reduced humidity in the desiccator. Furthermore, the high deviations of replicate experiments indicate that the ageing of pCS800 is the least predictable. The de-functionalization of the carbon surface during the high-temperature treatment in N_2_ creates reactive centers that are not saturated by N_2_ [30]. High-temperature treatment with H_2_ on the contrary not only de-functionalizes the carbon surface, but also saturates the resulting reactive carbon atoms and un-saturated aliphatic structures [18]. This procedure was recently applied in order to obtain AC felts with very low O-content and a positive surface charge, and proved to be suitable to generate excellent adsorbents for PFAS anions and other anionic persistent and mobile organic compounds (PMOCs) [31]. Most interestingly, the effect of the high-temperature treatment in 3 vol.-% H_2_O/N_2_ applied in this study was comparable to the treatment with H_2_. On the one hand, both treatment conditions, H_2_O/N_2_ as well as H_2_/N_2_ atmospheres, de-functionalized the surface more than the high-temperature treatment in pure N_2_. On the other hand, they both stabilized the surface afterwards, which resulted in a suppressed and thus slower and overall reduced ageing of the aCS surface. However, it is worth mentioning that the very low oxygen content of the samples treated in H_2_O/N_2_ as well as H_2_/N_2_ could not be completely maintained. Even upon storage in a desiccator, functional groups releasing CO_2_ during TPD were introduced presumably due to the chemisorption of O_2_. Nevertheless, the re-oxidation of the surface only occurred to a certain degree and could be maintained at a relatively low amount of surface oxygen.

**Figure 10:**
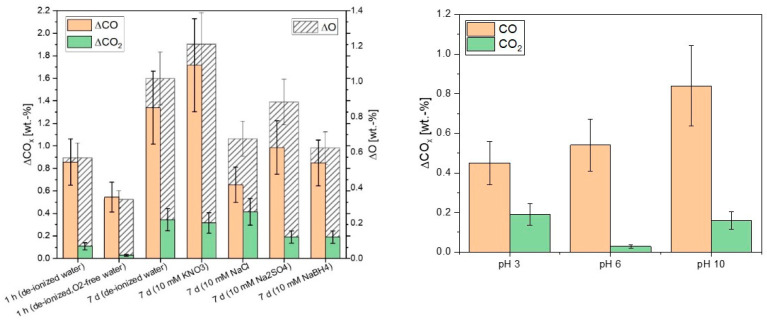
Increase of the CO- and CO_2_-releasing groups analyzed via TPD up to 1100 °C after ageing of aCS in different aqueous media (**left**) and in oxygen-free water for 1 h at three different pH values adjusted with HCl or NaOH (**right**). The given ΔCO_x_ values are the differences between fresh and aged samples. Error bars represent the mean deviation of single values from the mean value of at least two independent experiments.

Due to the planned application of the aCS in water, the according ageing mechanism was further investigated. In addition to the experiments in water shown in Figure 9 that were conducted in the presence of a limited amount of oxygen and for at least 7 d, experiments in oxygen-free water with exposure in shorter time-frames were performed. In Figure 10 (left), it is shown that the surface ageing monitored through the introduction of oxygen also occurred at very short ageing times of 1 h and even in oxygen-free water. Hence, it can be deduced that the major part of the observed ageing occurs upon the first contact with water due to the rapid saturation of reactive surface centers [30]. Specifically, half of the increase in the surface oxygen content of the sample that was aged for 7 d in water was already introduced after 1 h in water. The ageing in water was slightly enhanced or suppressed when different inorganic salts were added. KNO_3_ moderately enhanced the surface ageing, possibly due to a slight oxidizing effect of the nitrate and/or inducing a small change of pH. Sodium borohydride, in contrast, suppressed the oxygen-introduction due to its reducing effect. Additionally, sodium chloride and sodium sulfate showed a slightly suppressing effect on the introduction of oxygen on the aCS surface but had the opposite effect regarding the introduction of carboxylic and quinone/phenol groups, respectively. The ageing via the introduction of oxygen-containing groups could not be prevented in aqueous media as it was also observed in oxygen-free water. This means that there is a significant contribution of hydrolysis to the ageing process. In order to investigate the effect of hydrolysis on the aCS surface ageing, further experiments in O_2_-free water at different pH values were conducted. From Figure 10 (right), it can be seen that the introduction of CO_2_-releasing functionalities such as carboxylic groups is augmented at pH 3 as well as at pH 10. Therefore, it is assumed that these functionalities are introduced upon the hydrolysis of surface sites facilitated in acidic and alkaline media. Furthermore, it is noteworthy that the amount of CO-releasing groups is significantly enhanced only at pH 10. This could mean that, e.g., phenolic groups can be introduced by the reaction of hydroxide with reactive moieties on the aCS surface, presumably by a nucleophilic addition-like mechanism.

**Figure 11:**
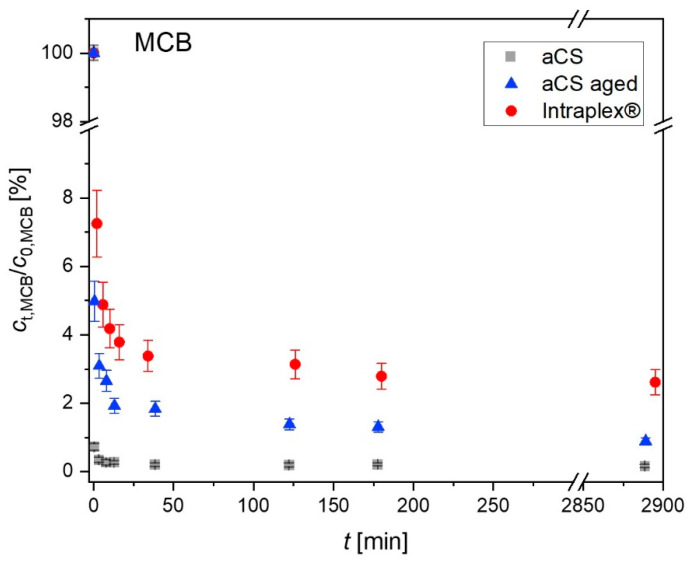
Time-resolved adsorption of MCB on aCS vs. aged aCS for MCB in comparison with the commercial finely ground AC sample Intraplex^®^. Error bars represent the minimum and maximum deviation from the mean value based on the experimental and analytical duplicates (i.e., n = 4). *c*_0,MCB_ = 2.5 mg L^−1^; *c*_aCS_ = 70 mg L^−1^; 10 mM KNO_3_; pH = 6.5.

The influence of the observed activated carbon ageing on the sorption behavior was characterized by monitoring the MCB sorption kinetics of freshly prepared aCS with an aged aCS sample in comparison with the commercial finely ground AC sample (Intraplex^®^) (Figure 11). The aged aCS sample was prepared by suspending the aCS sample in water and keeping it for 45 d in an abundant oxygen atmosphere. After this, “aged aCS” exhibited 4.6 wt.-% “TPD-oxygen”, meaning oxygen that is released as CO and CO_2_ upon heating up to 1100 °C. Note that this oxygen content is significantly higher than that of the above-shown aged samples (Figure 9).

In Figure 11, it can be seen that the three finely powdered AC samples show very fast adsorption kinetics as they all provide mean particle diameters of about 1 µm. The herein freshly prepared aCS adsorb MCB almost completely within the first minute. After the very fast initial adsorption, both aCS samples exhibit a slow pore diffusion due to the narrow microporous system. The equilibrium state is approached within 48 h. The equilibrium sorption coefficients log (*K*_D_/(L kg^−1^)) for MCB significantly decrease from aCS (7.2 ± 0.1) > aged aCS (6.6 ± 0.1) > Intraplex^®^ (5.9 ± 0.2). This correlates with the increase in the oxygen content of the samples from aCS (1.6 wt.-% O) < aged aCS (4.6 wt.-% O) < Intraplex^®^ (6.5 wt.-% O). The detailed TPD profiles can be viewed in the SI (Figure S1). The results of the kinetic evaluation according to Equations (3) and (4) are displayed in Table 3. The initial adsorption rate *r*_0_ was roughly estimated through the increase of the loading at the first sampling point of 30 s. However, due to the extremely fast initial adsorption, this parameter may be afflicted with a high error as the actual duration of the fast initial adsorption is unknown. The linear fitting for the estimation of the late-stage adsorption rate coefficient *k*_e_ (Equation (5)) is shown in Figure S4.

The film-mass-transfer coefficients *k*_f_ for the MCB adsorption on the three AC samples are essentially the same due to their comparable particle size. They are in the same order of magnitude as the literature results for the adsorption of toluene and phenol on adsorbent particles with radii of around 500 and 200 µm, respectively [32]. However, the k_f_ values in Table 3 seem to overestimate the thickness of the stagnant boundary layer around the small AC particles (*d*_film_ = *D*_MCB,water_/*k*_f_ = 10^−9^ m^2^ s^−1^/(1.3 × 10^−4^ m s^−1^) ≈ 10 µm). This may be due to the very short contact time of about 30 s, which does not ensure an instantaneous ideal mixing of the added MCB into the AC suspension. The surface diffusion coefficients *D*_s_ decrease from Intraplex^®^ > aCS > aged aCS and are several orders of magnitude smaller compared to the literature data [32,33]. This might be attributed to the predominant microporosity of the herein investigated adsorbents, which can impede fast surface diffusion processes [33]. This could also explain why the experimentally obtained *D*_s_ for Intraplex^®^ is an order of magnitude higher than that for the aCS samples. Intraplex^®^ provides larger micropores between 1 and 1.5 nm, which could facilitate intra-particle substance transport (cf. Figures S2 and S5).

**Figure 12:**
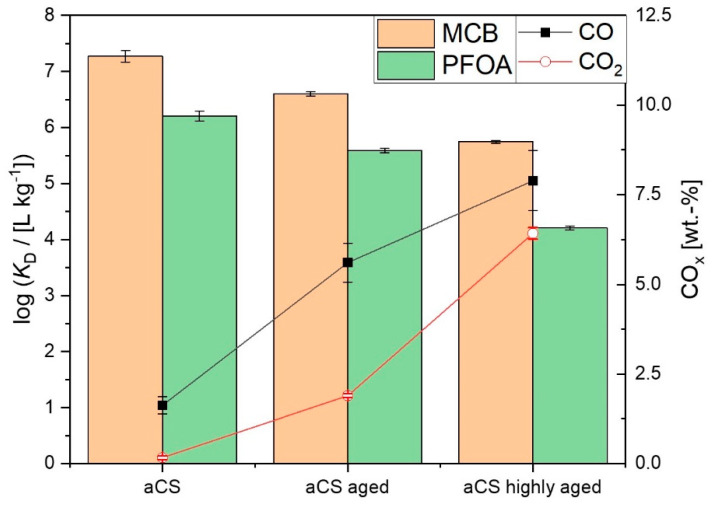
Adsorption coefficients log *K*_D_ on aCS vs. aged aCS for MCB (*c*_0,MCB_ = 2.5 mg L^−1^; *c*_aCS_ = 70 mg L^−1^; 10 mM KNO_3_; pH = 6.5) and PFOA (*c*_0,PFOA_ = 0.2 mg L^−1^; *c*_aCS_ = 200 mg L^−1^; 10 mM NaCl; pH = 6.5). Error bars represent the mean deviation of single values from the mean value of at least two independent experiments.

It was assumed that the ageing of aCS surfaces might have an even greater effect on the adsorption of PFOA than on the adsorption of MCB. Therefore, the adsorption affinity of freshly prepared aCS (1.6 wt.-% O) in comparison with aged aCS (4.6 wt.-% O) towards PFOA was monitored by means of single-point adsorption coefficients *K*_D_. In order to follow the observed trend, the adsorption on a highly aged sample (9.2 wt.-% O) was also investigated. The latter represents ageing in abundant oxygen for more than 8 months. The resulting log *K*_D_ values for the PFOA adsorption are displayed in Figure 12 in comparison with those for the MCB adsorption. As discussed above, the log *K*_D,MCB_ is significantly decreased for aged aCS compared to aCS. Regarding the adsorption of PFOA on “aged aCS”, the adsorption is decreased as well; however, not more than for the neutral MCB molecule. For the highly aged sample, the decrease of log *K*_D_ becomes slightly more pronounced for PFOA compared to MCB. At the experimental pH of 6.5, the aged adsorbents exhibit a negative net charge on the surface as the point of zero net charge (PZNC) decreases from pH = 7.0 ± 0.2 for aCS to pH ≤ 6.3 for the aged aCS samples. Accordingly, the decrease of adsorbent affinity towards PFOA with a decreasing PZNC due to electrostatic repulsion of the oxidized surface and the anionic PFOA molecule is in conformity with observed trends in the literature [34]. The adsorption affinity of an adsorbent towards PFOA is a complex interplay of the adsorbent characteristics such as charge, ion exchange capacities and various functional groups, but also a matter of pore size [16,27,35]. Nevertheless, the decrease in the adsorption affinity of the aged adsorbents towards MCB is also significant. These results highlight the need for monitoring adsorbent ageing and the investigation of the influence on the adsorption of different pollutant classes. Furthermore, it is necessary to avoid preventable oxidation during and directly after the synthesis of freshly de-functionalized surfaces. The herein proposed synthesis of aCS may be an alternative for either wet-grinding of AC or simple pyrolysis of hydrochar particles under N_2_, as both procedures can lead to enhanced surface oxidation.

## 3. Materials and Methods

### 3.1. Chemicals

Commercially available sugar was used as the starting material for the HTC synthesis. PFOA (96%) and CMC sodium salt with a molecular weight of 90 kDa, a polymerization degree of 400, a substitution degree of 0.65–0.90 with a sodium content of approximately 8 wt.-% and a purity of 99.5% was purchased from Sigma-Aldrich (St. Louis, MO, USA). MCB (≥99%), sodium chloride, sodium sulfate, sodium borohydride, sodium hydroxide, hydrochloric acid (37%) potassium hydroxide and potassium nitrate were all analytical grade and purchased from Merck (Darmstadt, Germany). Potassium hydrogen phthalate (p.a.) was purchased from Riedel-de Haën (Seelze, Germany).

### 3.2. Preparation of Activated Carbon Spheres

Activated carbon spheres were prepared from hydrothermal carbonaceous spheres (CS). The synthesis was described in our previous work [12]. In brief, 0.5 M sucrose (171.2 g L^−1^) dissolved in de-ionized water with 1 wt.-% of CMC (in relation to the initial amount of sucrose) was placed in a stainless steel autoclave with a glass insert and heated in an oven for 2 h at 180 °C. The formed precipitate was separated from the liquid phase through centrifugation, washed once with de-ionized water and then air-dried. CS were pyrolyzed in a horizontal quartz reactor tube with a continuous N_2_ gas flow of 200 mL min^−1^ under various conditions. Initially, the heating rate was varied between 100, 10 and 1 K min^−1^. If not stated otherwise, the latter was used for all pyrolysis experiments in order to ensure an optimal dispersibility of the product. CS pyrolyzed (pCS) under N_2_ at 400, 600 and 800 °C for 1 h were named pCS400, pCS600 and pCS800, respectively. The addition of “-H_2_O” after the temperature indicates pyrolysis in a 3 vol.-% H_2_O/N_2_ mixture. For this, the dry nitrogen flow passed a water reservoir in order to allow water uptake into the gas flow at room temperature (23 ± 2) °C before it entered the pyrolysis reactor. If the pyrolysis time deviated from 1 h, this was indicated at the end of the respective sample names (“pCS800-H_2_O-2h” and “pCS800-H_2_O-4h”). CS treated under H_2_O/N_2_ at 800 °C for 4 h were called aCS.

### 3.3. Ageing of Activated Carbon Spheres

The ageing of aCS was conducted under various conditions: in a laboratory air atmosphere, in a desiccator with silica gel as a drying agent and suspended in different aqueous media (de-ionized water and salt solutions containing 10 mM KNO_3_, NaCl, Na_2_SO_4_ or NaBH_4_, respectively). The surface oxygen content of the aCS was analyzed via TPD directly after the preparation and after storage for various time periods. The ageing in air/water of aCS was compared with two differently prepared samples: pCS800 (pyrolyzed at 800 °C in N_2_) and aCS-H_2_ for which aCS were post-treated at 900 °C in 10 vol.-% H_2_/N_2_.

For the ageing in air, 50 mg of the samples were stored in open beakers under a laboratory atmosphere or in the desiccator (not evacuated), respectively. The ageing in water was conducted with sample amounts of 15 mg in closed vials with 25 mL oxygen-containing de-ionized water and 5 mL air in the headspace. They were continuously shaken on a horizontal shaker with 180 rpm during the defined ageing time. For experiments in oxygen-free water, the water-containing vials were flushed with N_2_ for 20 min prior to the addition of aCS and 10 min afterwards before closing the vials until airtight and shaking them likewise. After defined periods of ageing time, the aCS samples were transferred to a tubular furnace and dried at 100 °C in an N_2_-flow.

### 3.4. Adsorption Experiments

The synthesized aCS were prepared for sorption experiments by shaking 100–200 mg L^−1^ of aCS in a salt solution (10 mM KNO_3_ or 10 mM NaCl) at (23 ± 2) °C for at least 24 h at 180 rpm. After that, the pH of the mixture was checked and, if necessary, adjusted to 6.5 with 0.1 M NaOH or HCl. The aCS were then fully dispersed by ultrasonication for 10 min and spiked with the according amount of stock solutions of the pollutants (MCB in acetone, PFOA in 10 mM aqueous NaCl). After 1 min of vigorous shaking by hand, the spiked suspensions were continuously shaken on a horizontal shaker with 180 rpm. MCB was monitored using gas chromatography coupled with mass spectrometry (GC-MS) through manual sampling of the batch headspace with a gas-tight syringe. For PFOA analysis, aliquots of the aqueous suspensions were taken, filtered through 0.45 µm cellulose acetate filters and afterwards diluted with methanol and subsequently analyzed via liquid chromatography coupled with mass spectrometry (LC-MS, see below). For kinetic experiments, headspace or aqueous samples were taken after defined periods of time and analyzed accordingly.

The thermodynamic data of MCB adsorption were evaluated according to the Langmuir model with the software OriginPro 2018G (© 1991–2017 OriginLab Corporation, Northampton, MA, USA). For fitting the MCB adsorption on the different pCS samples, a linearized form of the Langmuir Equation (1) was used.

(1) $$q_{e}=q_{m}-\left[\frac{1}{K_{L}}\right]\frac{q_{e}}{c_{e}}$$

where *q*_m_ [mg g^−1^] is the maximum monolayer loading of MCB on the adsorbent, and *c*_e_ [mg L^−1^] and *q*_e_ [mg g^−1^] are the equilibrium sorbate concentration and loading, respectively. *K*_L_ [L mg^−1^] is the Langmuir constant, which is a measure of the affinity between the adsorbent and adsorbate.

For evaluation of the PFOA adsorption, the linearized form of the Freundlich model (Equation (2)) was used.

(2) $$\log q_{e}{=n}^{-1}\cdot \log c_{e}+\log K_{F}$$

where *q*_e_ [mg kg^−1^] and *c*_e_ [mg L^−1^] are the equilibrium loading and dissolved concentration of PFOA. The Freundlich constant *K*_F_ [(mg kg^−1^)/(mg L^−1^)^1/*n*^] is a measure of the adsorption affinity and *n^−^*^1^ is the Freundlich exponent, which expresses the deviation of the isotherm from a linear correlation.

Single point adsorption coefficients *K*_D_ [L kg*^−^*^1^] were determined as ratios *q*_e_/*c*_e_.

The kinetic evaluation of time-resolved adsorption data was performed according to the method described by Yao et al. [32].

The film mass transfer coefficient *k*_f_ [m s*^−^*^1^] was estimated based on the adsorbent particle radius *R* [m], the adsorbent particle density of the air-filled particles *ρ*_p_ [g L*^−^*^1^], the initial adsorbate concentration *c*_0_ [mg L*^−^*^1^] and the initial adsorption rate *r*_0_ = d*q*_t_/d*t* [mg g*^−^*^1^ s*^−^*^1^] (Equation (3)). In the present work, the latter was defined as the initial change in the loading *q* between *t* = 0 and 0.5 min.

(3) $$k_{f}=\frac{{R\rho }_{P}r_{0}}{{3c}_{0}}$$

The surface diffusion coefficient *D*_s_ [m^2^ s^−1^] can be obtained via Equation (4):

(4) $$D_{s}=\frac{{R/\pi }^{2}}{\frac{1}{k_{e}}\left[1+\frac{m}{V}{\left(\frac{dq}{dc}\right)}_{e}\right]\;-\;\frac{{R\rho }_{P}}{{3k}_{f}}{\left(\frac{dq}{dc}\right)}_{e}}$$

where *m*/*V* is the dosage of adsorbent [g L^−1^], (d*q*/d*c*)_e_ [L g^−1^] is the slope of the adsorption isotherm at the respective equilibrium loading and *k*_e_ [s^−1^] is the late stage first-order adsorption rate coefficient. The estimation of *k*_e_ was carried out through the following method:

(5) $$-\ln\left(1-\frac{q_{t}}{q_{e}}\right){=b+k}_{e}t$$

After plotting −ln(1 − *q*_t_/*q*_e_) versus *t*, *k*_e_ can be obtained from the slope of the linear regression line. *b* represents an integration constant. Equation (5) is only used in the late adsorption stage close to the equilibrium state [32].

### 3.5. Analytical Methods

Optical microscopic images of particles and suspensions were recorded with a VHX digital microscope (Keyence, Neu-Isenburg, Germany). Random samples of 100–150 particles were measured with the open-source software ImageJ 1.52a [36] in order to determine the particle diameters for the dry CS samples. The measured values were processed with RStudio 1.4 [37] in order to obtain the frequency distribution of the particle size.

The scanning electron microscopy (SEM) analyses were conducted with a Zeiss Merlin VP compact microscope. The beam current was 250 pA and the electron landing energy added up to 10 kV.

The C-contents of the dry CS samples were determined either with a TruSpec^®^ CHN macro-analyzer (LECO, St. Joseph, MI, USA) or with a C-Mat 5500 (Ströhlein Instruments, Germany).

For analysis of the total organic carbon (TOC) concentration in particle suspensions, the diluted aqueous samples were injected via a syringe into a two-zone combustion unit consisting of a thermal combustion zone (780 °C) and a catalytic post-oxidation zone containing a Pt/Al_2_O_3_ catalyst (700 °C). The samples were fully oxidized and the resulting gas stream was dried with a membrane dryer. CO_2_ was detected with a nondispersive infrared detector. Potassium hydrogen phthalate was used as the calibration standard.

The zeta potential as well as the particle size distribution of the samples in aqueous suspension were characterized with a Zetasizer Ultra (Malvern Panalytical, Kassel, Germany). The samples were prepared by dispersing the aCS in 10 mM KNO_3_ at a concentration of 5 mg L^−1^ and the pH was adjusted to 6 with 1 M KOH.

TPD was performed under Ar (50 mL min^−1^) with a BELCAT-B chemisorption analyzer (BEL). The samples were pretreated at 50 °C for 30 min, then heated to 1100 °C with 10 K min^−1^ and held for 30 min. Evolving CO and CO_2_ were detected with an Infralyt detector (SAXON Junkalor, Dessau-Roßlau, Germany).

The specific surface area as well as the pore-size distribution were determined from CO_2_ ad- and desorption isotherms measured at 273 K by using the Software ASiQwin (Version 5.0) and included a density functional theory (DFT) model (non-local DFT for CO_2_ sorption at 273 K in carbon slit pore, equilibrium model). Prior to the data evaluation, the CO_2_ ad- and desorption isotherms at 273 K were measured with the manometric sorption analyzer AUTOSORB-iQ (Quantachrome Instruments, Boynton Beach, FL, USA). For temperature control, a cryoTune (3P Instruments GmbH & Co. KG, Leipzig, Germany) was used. Before starting the measurement, about 50–100 mg of the carbon material was transferred to the measuring cell and then pre-treated for 10 h at 423 K and a final vacuum of <10^−2^ Pa. Subsequently, the sample was weighed again, the activated sample amount was determined and the measurement started. The measurement began with the determination of the void volume with He (Air Products, Lüneburg, Germany, Purity 5.2; 99.9992%) at ambient temperature (298 K). After that, vacuum was applied again for approx. 30 min, the temperature was lowered to 273 K and the actual measurement with CO_2_ (Air Products, Lüneburg, Germany, purity 4.5; 99.995%) was started. The equilibration times were set to at least 8 min per step. The generated measurement data were automatically converted to a volume-based loading of carbon dioxide per gram of carbon material as a function of the detected absolute pressure at the constant temperature of 273 K. The mean pore diameter represents the pore width at one half of the total pore volume.

Headspace GC-MS samples were injected into a GCMS-QP2010 (Shimadzu, Jena, Germany) at 180 °C with a column temperature of 140 °C and a He flow of 1 mL min^−1^. A DB-5ms column (Agilent, 30 m × 0.25 mm × 0.25 µm) was used.

LC-MS measurements were carried out with a LCMS-2020 (Shimadzu, Jena, Germany) equipped with a Gemini C6-Phenyl column (100 mm × 2 mm, Phenomenex, Aschaffenburg, Germany, with a particle size of 3 µm and pore size of 110 Å) at a column temperature of 40 °C. The mobile phase consisted of a mixture of 35 vol.-% solvent A (20 mM ammonium acetate in a 9:1 mixture of de-ionized water and methanol) and 65 vol.-% solvent B (20 mM ammonium acetate in a 9:1 mixture of methanol and de-ionized water) and was delivered at a flow rate of 0.25 mL min^−1^.

## 4. Conclusions

Uniform and dispersible aCS were synthesized bottom-up via hydrothermal carbonization of sucrose with subsequent pyrolysis. The dosage of 3 vol.-% H_2_O in N_2_ flow led to high specific surface areas up to 1400 m^2^ g^−1^ with a well-pronounced microporosity and a highly de-functionalized surface, which was moderately stable against hydrolysis and oxidation at ambient conditions. Pyrolysis with 3 vol.-% H_2_O/N_2_ could replace 10 vol.-% H_2_/N_2_ for the generation of sufficiently stable, hydrophobic carbonaceous adsorbent surfaces. However, a certain degree of re-oxidation could not be prevented for all investigated samples, even the ones where the surface was saturated with H_2_ after de-functionalization. This has to be kept in mind, especially when adsorbents are characterized in the dry state and then applied in water. A comparative experiment where the enhanced surface ageing of the adsorbent was induced in water with abundant oxygen showed that the aCS suffered a significant loss regarding their sorption affinity, even when it comes to the neutral MCB molecule. This negative effect becomes even stronger for negatively charged adsorbates such as PFOA. Therefore, we attempted to even out the decreases regarding the sorption performance upon inevitable surface ageing in water by adjusting the pore system of the synthesized aCS. The very narrow and strictly microporous system performed well regarding the PFOA adsorption with adsorption coefficients up to 10^6.5^ L kg^−1^ at *c*_e,PFOA_ ≈ 1 µg L^−1^. Future studies will deal with up-scaling of the developed synthesis process and elucidate the adsorption performance of aCS in more complex scenarios, e.g., with mixed contaminants and dissolved natural organic matter.

## Figures and Tables

**Table 1:** Summary of CS samples pyrolyzed under different conditions along with their specific surface areas derived from CO_2_ ad-/desorption (density functional theory (DFT) method) and their C-contents determined by combustion. The error ranges were derived from the relative mean deviation of single values from the mean value of at least two independent experiments.

Treatment Conditions (Gas Atmosphere, Final Temperature, Heating Rate and Holding Time)					
Fixed Parameters	Varied Parameter	Sample Name	Surface Area by Applying DFT Method [m^2^ g^−1^]	Mean Pore Diameter [nm]	Carbon Content [wt.-%]
1 K min^−1^, N_2_, 1 h	400 °C	pCS400	386 ± 5	0.60 ± 0.01	78 ± 1
	600 °C	pCS600	653 ± 8	0.58 ± 0.01	88 ± 1
	800 °C	pCS800	877 ± 10	0.56 ± 0.01	92 ± 2
1 K min^−1^, H_2_O/N_2_, 1 h	800 °C	pCS800-H_2_O	954 ± 11	0.58 ± 0.01	96 ± 2
	840 °C	pCS840-H_2_O	1178 ± 14	0.63 ± 0.01	98 ± 2
	880 °C	pCS880-H_2_O	1386 ± 17	0.68 ± 0.01	96 ± 2
1 K min^−1^, H_2_O/N_2_, 800 °C	2 h	pCS800-H_2_O-2h	1031 ± 12	0.60 ± 0.01	87 ± 2
	4 h	pCS800-H_2_O-4h = aCS	1015 ± 12	0.60 ± 0.01	91 ± 2
1 K min^−1^, H_2_O/N_2_, 800 °C, 4 h	post-treatment at 900 °C, 2 h, 10 vol.-% H_2_/N_2_	aCS-H_2_	1030 ± 12	0.61 ± 0.01	92 ± 2

**Table 2:** Fitting parameters for the evaluation of the experimentally obtained sorption isotherms for monochlorobenzene (MCB) according to the linearized Langmuir Equation (1) and compared with the experimentally obtained maximum loading (*q*_max_ (exp) derived from at least two replicate experimental data points). The error ranges of the experimental values represent the mean deviation of single values from the mean value of at least two independent experiments. The error ranges of the fitted parameters were derived from the standard error of regression via propagation of uncertainty.

Sample Name	*q*_max_ (exp)	*q*_max_ (fit)	*K*_L_	*R* ^2^
	[mg g^−1^]	[mg g^−1^]	[L mg^−1^]	
**pCS400**	20 ± 1	32 ± 5	1.0 ± 0.3	0.81
**pCS600**	104 ± 3	104 ± 4	1.2 ± 0.2	0.95
**pCS800**	106 ± 2	119 ± 3	1.3 ± 0.1	0.97
**pCS800-H_2_O**	171 ± 1	171 ± 5	2.0 ± 0.3	0.94
**pCS840-H_2_O**	169 ± 4	168 ± 4	2.1 ± 0.2	0.95
**pCS880-H_2_O**	204 ± 3	194 ± 7	3.6 ± 0.6	0.89
**aCS**	196 ± 6	193 ± 5	3.0 ± 0.4	0.92

**Table 3:** Derived parameters *r*_0_ and *k*_e_ for the estimation of the film mass transfer (*k*_f_) and surface diffusion (*D*_s_) coefficients of the MCB adsorption on the different activated carbon samples according to the method proposed in [32].

AC Sample	*r*_0_ [mg g^−1^ s^−1^]	*k*_e_∙10^4^ [s^−1^]	*k*_f_∙10^4^ [m s^−1^]	*D*_s_∙10^20^ [m^2^ s^−1^]
**aCS**	1.2	7.9	1.3	2.3
**Aged aCS**	1.3	5.4	1.4	1.0
**Intraplex^®^**	1.2	1.1	1.3	25.7

## Data Availability

The data presented in this study are available on request from the corresponding author.

## Supplementary Materials

The following supporting information can be downloaded at: https://www.mdpi.com/article/10.3390/ijms24043831/s1.
