# Supplementary material for: Bottom-Up Synthesis of De-Functionalized and Dispersible Carbon Spheres as Colloidal Adsorbent
Source: Int J Mol Sci. 2023 Feb 14;24(4):3831. doi: 10.3390/ijms24043831 (PMC9964220; doi:10.3390/ijms24043831)
Supplement: Supplementary file 1 [file ijms-24-03831-s001.zip › ijms-2221597-supplementary.pdf]

Supporting Information

Bottom-up synthesis of de-functionalized and dispersible carbon spheres as colloidal adsorbent

Maria Balda [1], Katrin Mackenzie [1] , Silke Woszidlo [1], Hans Uhlig [2], Jens Möllmer [2], Frank-Dieter Kopinke [1], Gerrit Schüürmann [3,4] and Anett Georgi [1,*]

[1] Department of Environmental Engineering, Helmholtz Centre for Environmental Research – UFZ, 04318 Leipzig, Germany
[2] Institut für Nichtklassische Chemie e.V. – INC, 04318 Leipzig, Germany
[3] Institute of Organic Chemistry, Technical University Bergakademie Freiberg, 09599 Freiberg, Germany
[4] Department of Ecological Chemistry, Helmholtz Centre for Environmental Research – UFZ, 04318 Leipzig, Germany
* Correspondence: anett.georgi@ufz.de

5 Figures

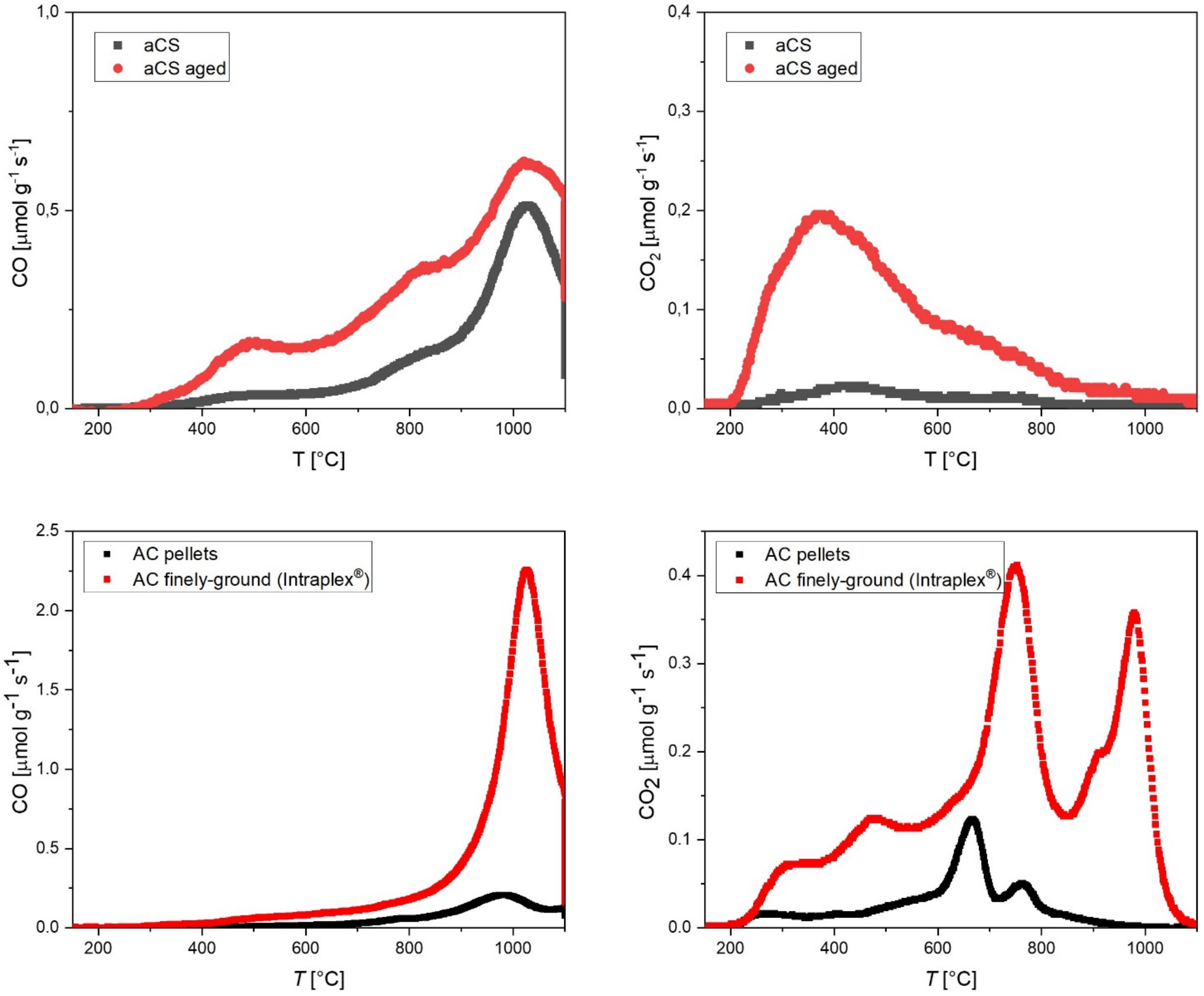

Figure S1. Temperature-programmed decomposition (TPD) up to 1100 °C (10 K min^{-1}) of activated carbon spheres (aCS) compared with aCS aged (top) and a commercial activated carbon (AC) sample Intraplex® obtained by a wet-milling process ("AC finely-ground") compared with the respective precursor material in pellet-form ("AC pellets")

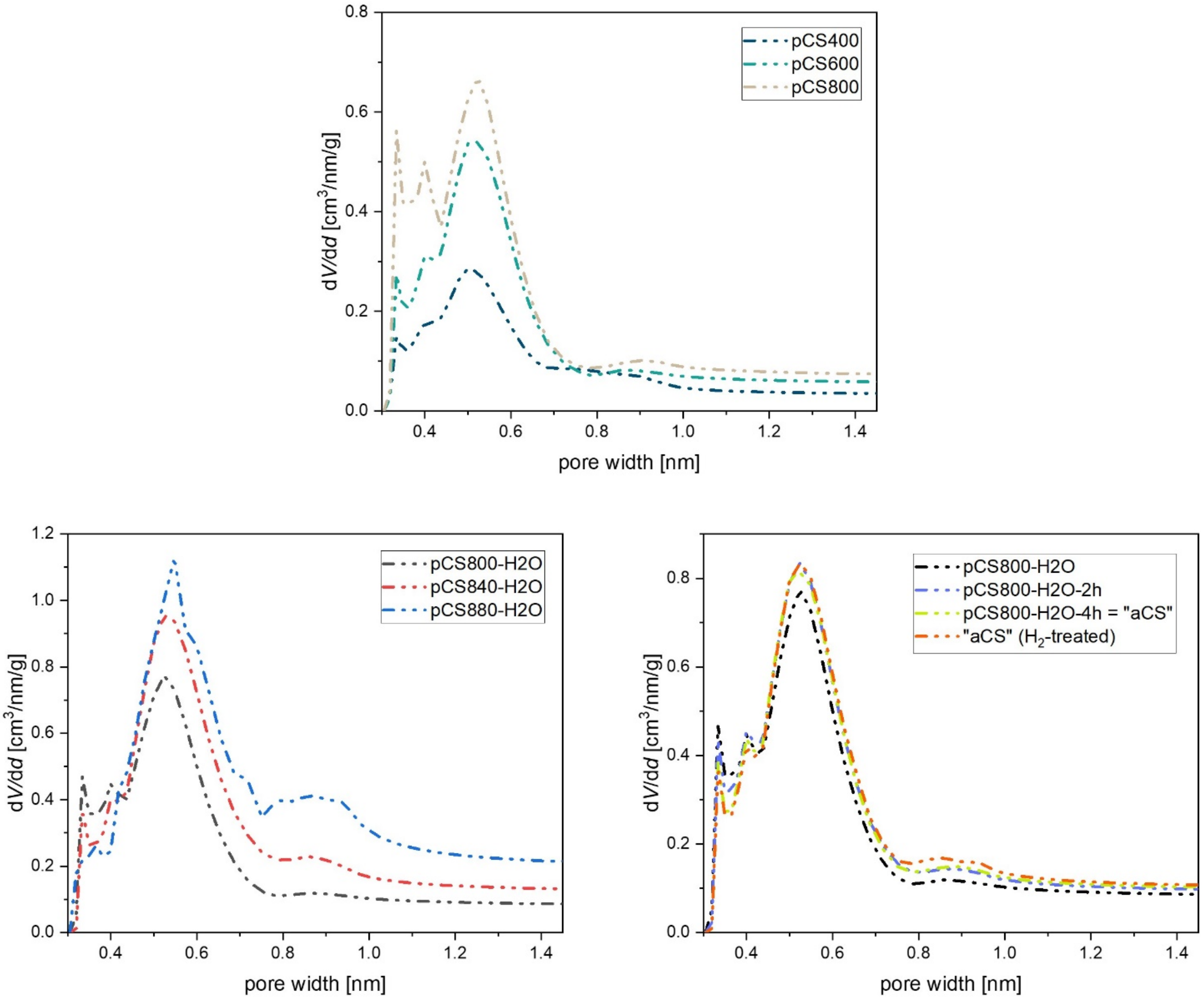

Figure S2. Pore size distributions for all pyrolyzed and activated CS samples determined with NLDFT based on CO_2 ad- and desorption (isotherms measured up to p/p_0 = 0.0287 equals to p = 0.1 MPa).

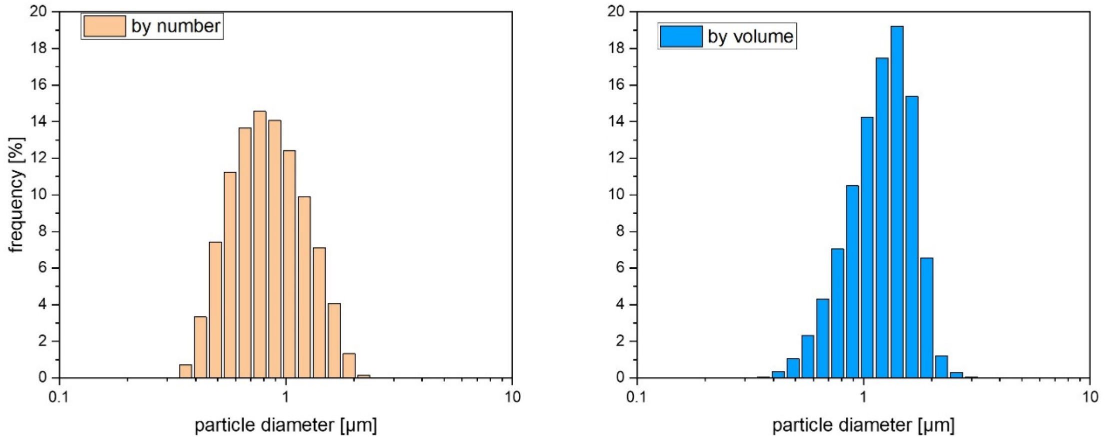

Figure S3. Differential particle size distribution by number and by volume determined *via* dynamic light scattering (DLS) analysis of 5 mg L^{-1} aCS particles dispersed in 10 mM KNO_3 solution at pH = 6.

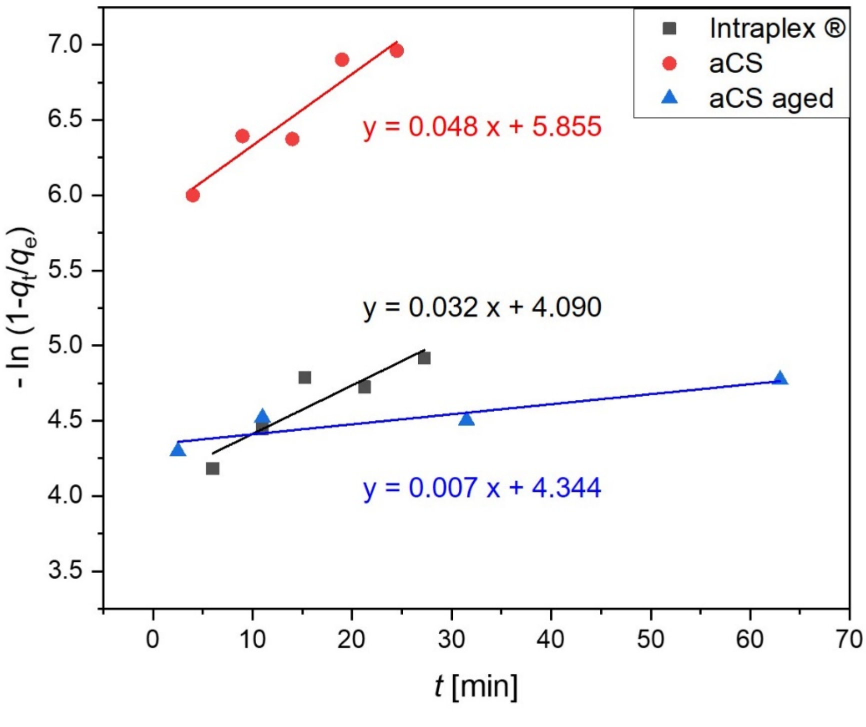

Figure S4. Linear fitting of the kinetic data of the monochlorobenzene (MCB) adsorption according to Equation 5 for estimation of the late stage adsorption rates of MCB on the different activated carbon samples.

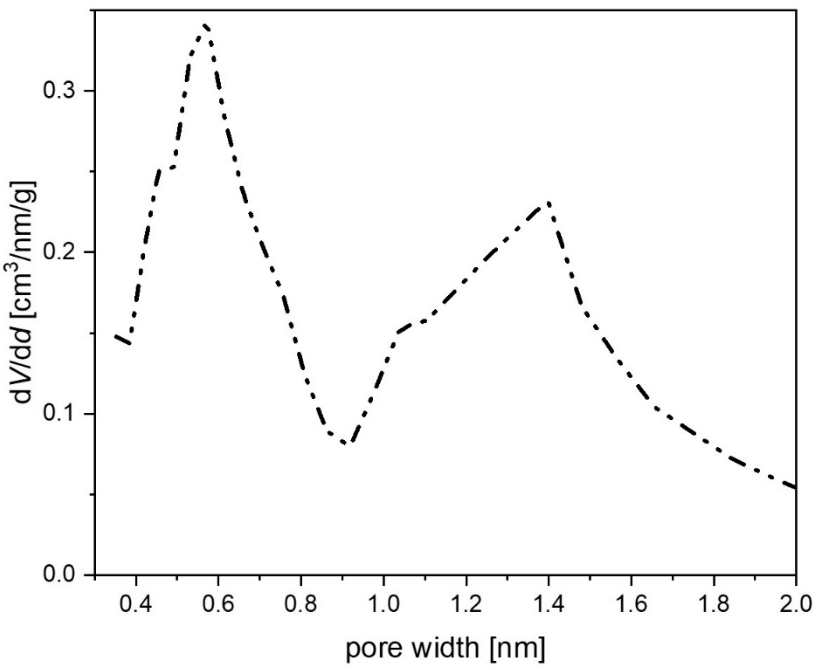

Figure S5. Pore size distribution of the AC Intraplex® determined with NLDFT based on CO_2 adsorption (up to $p/p_0 = 1$, with $p_0 = 3.4851$ MPa).
